# 

**Published:** 1903-06

**Authors:** 


### The Library of the Bristol Medico-Chirurgical Society.

*The following donations have been received since the publication of the List in March :*

*May 30th, 1903.*

| | |
|---|---|
| J. Paul Bush, C.M.G. (1) ... ... ... ... ... | 1 volume. |
| L. M. Griffiths (2) ... ... ... ... ... ... ... | 6 volumes. |
| The Laryngological Society of London (3) ... ... | 1 volume. |
| The Scholastic Trading Co. (4) ... ... ... ... | 2 volumes. |
| R. Shingleton Smith, M.D. (5) ... ... ... ... | 1 volume. |
| James Swain, M.D. (6) ... ... ... ... ... ... | 1 volume. |

### FORTY-EIGHTH LIST OF BOOKS.

The titles of books mentioned in previous lists are not repeated.

The figures in brackets refer to the figures after the names of the donors, and show by whom the volumes were presented. The books to which no such figures are attached have either been bought from the Library Fund or received through the *Journal*.

**Allingham, H. W.** *Operative Surgery* ... ... ... ... ... ... ... ... ... ... 1903

**Benham, P. Brouardel and F. L.** *Death and Sudden Death* ... 2nd Ed. 1902

**Bridge, N.** ... ... *Tuberculosis* ... ... ... ... ... ... ... ... ... ... 1903

**Brouardel and F. L. Benham, P.** *Death and Sudden Death* ... 2nd Ed. 1902

**Burghard, W. W. Cheyne and F. F.** *A Manual of Surgical Treatment.* Part VI. Section 2 ... ... ... ... ... ... ... 1903

**Calvert, J.** ... ... *Practical Pharmacy and Prescribing* ... ... 2nd Ed. 1903

**Campbell, K.** ... *Refraction of the Eye* ... ... ... ... ... ... ... ... 1903

**Cheyne and F. F. Burghard, W. W.** *A Manual of Surgical Treatment* Part VI. Section 2 ... ... ... ... ... ... ... 1903

**Clemow, F. G.** ... *The Geography of Disease* ... ... ... ... ... ... ... 1903

**D'Estrées, D.** ... *Thirty-five years at Contrexéville.* (Tr. by A. C. Grylls) 1903

**Dowse, T. S.** ... *The Pocket Therapist* ... ... ... ... ... 3rd Ed. 1903

**Eccles, W. McA.**... *The Imperfectly Descended Testis* ... ... ... ... ... ... 1903

**Fagge and P. H. Pye-Smith, C. H.** *A Text-book of Medicine.* 2 vols. 4th Ed. 1901–02

**Fox, E. L.** ... ... *Only a Doctor* ... ... ... ... ... ... ... ... ... ... (2) 1902

*France, Index médical des principales Stations Thermales de* ... ... ... (1) 1903

**Froussard, [—]** ... *Muco-Membranous Colitis* ... ... ... ... ... ... ... 1903
**Grünwald, L.** ... *Atlas and Epitome of Diseases of the Mouth, Pharynx and Nose.* (Translated. Ed. by J. E. Newcomb) ... ... ... ... ... ... ... 2nd Ed. 1903
**Haines, F. Peterson and W. S.** [Eds.] *A Text-book of Legal Medicine and Toxicology.* Vol. I. ... ... ... ... ... ... 1903
**Jenner, G. C.** ... *Evidence at large as laid before the House of Commons respecting Vaccine Inoculation* ... ... ... ... 1805
**Kenwood, H. R.**... *Public Health Laboratory Work* ... ... ... 3rd Ed. 1903
**Lawson, G.** ... ... *Diseases and Injuries of the Eye.* (Ed. by A. Lawson) 6th Ed. 1903
*Library of the Royal College of Physicians of London, Accessions to the*... 1901-02
**Macleod, J. M. H.** *Handbook of the Pathology of the Skin*... ... ... ... 1903
**Manson, P.** ... ... *Tropical Diseases* ... ... ... ... ... ... [3rd Ed.] 1903
**Molière, [J. P. B.]** *Le Malade Imaginaire.* (With Notes by A. E. Ragon) (2) New Ed. 1889
**Morris, M.** ... ... *Diseases of the Skin* ... ... ... ... ... New [3rd] Ed. [1903]
**Noorden, C. von**... *Disorders of Metabolism and Nutrition.* (Ed. by Boardman Reed.) Parts I.—III. ... ... ... ... ... 1903
**Norström, G.** ... *Chronic Headache and its Treatment by Massage* ... ... 1902
,, *The Manual Treatment of Diseases of Women* ... ... 1903
**Pawlow, J. P.** ... *The Work of the Digestive Glands.* (Tr. by W. H. Thompson) ... ... ... ... ... ... ... ... ... 1902
**Peterson and W. S. Haines, F.** [Eds.] *A Text-book of Legal Medicine and Toxicology.* Vol. I. ... ... ... ... ... ... 1903
**Proust, R.** ... ... *Manuel de la Prostatectomie perinéale pour Hypertrophie* 1903
**Pye-Smith, C. H. Fagge and P. H.** *A Text-book of Medicine.* 2 vols. 4th Ed. 1901-02
**Richer, P.** ... ... *L'Art et la Médecine* ... ... ... ... ... ... ... ... [N.D.]
**Ross, R.** ... ... *Report on Malaria at Ismailia and Suez* ... ... ... 1903
**Sajous, C. E. de M.** *The Internal Secretions and the Principles of Medicine* Vol. I. 1903
**Scheube, B.**... ... *The Diseases of Warm Countries.* (Tr. by Pauline Falcke. Ed. by J. Cantlie) ... ... 2nd Ed. 1903
**Sobotta, J.** ... ... *Atlas and Epitome of Human Histology* (Translated. Ed. by G. C. Huber)... ... ... ... ... ... 1903
**Tunstall, F. J. Warwick and A. C.** *"First Aid" to the Injured and Sick.* 3rd Ed. 1903
**Wallace, J. S.** ... *The Physiology of Mastication* ... ... ... ... ... 1903
**Warwick and A. C. Tunstall, F. J.** *"First Aid" to the Injured and Sick.* 3rd Ed. 190
**Williams, J. W.**... *Obstetrics* ... ... ... ... ... ... ... ... ... ... 1903

### TRANSACTIONS, REPORTS, JOURNALS, &c.

Alienist and Neurologist, The... ... ... ... ... ... ... ... Vol. XXIII. 1902
American Dermatological Association, Transactions of the, for 1902 ... 1903
American Gynecology ... ... ... ... ... ... ... ... ... Vol. I. 1902

American Journal of Medical Sciences, The ... ... (5) Vol. CXXIII. 1902

American Journal of Obstetrics, The ... ... ... ... ... Vol. XLVI. 1902

American Laryngological Association, Transactions of the ... ... ... 1902

American Medicine ... ... ... ... ... ... ... ... ... ... Vol. IV. 1902

American Ophthalmological Association, Transactions of the Vol. IX. Part 3 1902

American Surgical Association, Transactions of the... ... ...Vol. XX. 1902

American Year-Book of Medicine and Surgery ... ... ... ... ... 1901-03

Archives of Pediatrics ... ... ... ... ... ... ... ... Vol. XIX. 1902

Archives provinciales de Chirurgie ... ... ... ... ... Tome XI. 1902

Australian Medical Gazette ... ... ... ... ... Vols. XX., XXI. 1901-02

Bookseller, The ... ... ... ... ... ... ... ... ... ... ... ... ... 1902

Boston Medical and Surgical Journal, The... ... ... Vol. CXLVII. 1902

Bristol Medico-Chirurgical Journal, The ... ... ... ... ...Vol. XX. 1902

British Journal of Dermatology, The ... ... ... ... ... Vol. XIV. 1902

Brooklyn Medical Journal, The ... ... ... ... ... ... Vol. XVI. 1902

Bulletin de l'Académie de Médecine ... ... Tom. XLVII., XLVIII. 1902

Bulletin de l'Académie royale de Médecine de Belgique ... Tome XVI. 1902

Bulletin de l'Institut international de Bibliographie ... ... ... 1899-1901

Bulletin of the Association of Medical Librarians ... ... ... Vol. I. 1902

Catalogue of Books for 1902, The English ... ... ... ... ... ... ... 1903

Centralblatt für Chirurgie ... ... ... ... ... ... ... ... ... ... 1902

Centralblatt für Gynäkologie ... ... ... ... ... ... ... ... ... ... 1902

China Medical Missionary Journal, The ... ... ... ... Vol. XVI. 1902

Cincinnati Lancet-Clinic, The ... ... ... ... ... Vol. LXXXVIII. 1902

Epidemiological Society of London, Transactions of the... Vol. XXI. 1902

,, ,, ,, ,, ,, Index, 1855-1900 [N.D.]

Gazette des Hôpitaux ... ... ... ... ... ... ... ... ... ... ... ... 1902

Gazette des Hôpitaux de Toulouse ... ... ... ... ... ... ... ... 1902

Gazette hebdomadaire des Sciences médicales de Bordeaux Tome XXIII. 1902

Gazette médicale de Paris ... ... ... ... ... ... ... ... ... ... ... 1902

Grèce médicale, La ... ... ... ... ... ... ... ... ... ... Vol. IV. 1902

Hospitals—

Boston City Hospital, Medical and Surgical Reports of the. (6) 13th series 1902

Middlesex Hospital Reports for 1901, The ... ... ... ... ... 1903

Indian Medical Gazette, The ... ... ... ... ... ... ... Vol. XXXVII. 1902

Intercolonial Medical Journal of Australasia ... ... ... Vol. VII. 1902

International Medical Magazine, The ... ... ... ... ... ... Vol. XI. 1902

Johns Hopkins Hospital Bulletin ... ... ... ... ... ... Vol. XIII. 1902

Journal d'Hygiène ... ... ... ... ... ... ... ... ... ... ... 1901-02

Journal of Balneology and Climatology, The ... ... ... ... Vol. VI. 1902

Journal of Hygiene, The ... ... ... ... ... ... ... ... ... Vol. II. 1902

Journal of Medical Research, The ... ... ... ... ... ... Vol. VIII. 1902

Journal of Mental Pathology, The ... ... ... ... ... ... ... Vol. II. 1902

Journal of Mental Science, The ... ... ... ... ... ... Vol. XLVIII. 1902

Journal of Nervous and Mental Disease, The ... ... ... Vol. XXIX. 1902

Journal of Tropical Medicine, The ... ... ... ... ... ... ... Vol. V. 1902

Journal of the American Medical Association, The ... ...Vol. XXXIX. 1902

Laboratory Reports of the Royal College of Physicians of Edinburgh. Vol. VIII. 1903

Laryngological Society of London, Proceedings of the ... (3) Vol. IX. 1902

Library Association Year-Book, The ... ... ... ... ... ... ... ... .. 1903

Medical Age, The ... ... ... ... ... ... ... ... ... Vol. XX. 1902

Medical Annual, The... ... ... ... ... ... ... ... ... ... ... ... 1903

Medical News, The ... ... ... ... ... ... ... ... ...Vol. LXXXI. 1902

Medical Review, The ... ... .. ... ... ... ... ... ... Vol. V. 1902

Medical Review of Reviews, The ... ... ... ... ... ... Vol. VIII. 1902

Merck's Annual Report for 1902 ... ... ... ... ... ... ... ... ... 1903

Mercy and Truth ... ... ... ... ... ... ... ... ... ... Vol. VI. 1902

Monthly Cyclopædia of Practical Medicine, The ... ... ... Vol. V. 1902

Montreal Medical Journal, The ... ... ... ... ... ... Vol. XXXI. [1902]

New York Medical Journal, The ... ... ... ... ... ...Vol. LXXVI. 1902

New Zealand Medical Journal, The ... ... ... ... ... N.S., Vol. I. 1901

Occidental Medical Times ... ... ... ... ... ... ... Vol. XVI. 1902

Ohio State Medical Society, Transactions of the ... ... ... ... ... 1902

Philadelphia County Medical Society, Proceedings of the Vol. XXII. 1901

Philadelphia Medical Journal, The ... ... ... ... ... ... Vol. X. 1902

Polyclinic, The ... ... ... ... ... ... ... ... ... ... ... Vol. VI. 1902

Post-Graduate, The ... ... ... ... ... ... ... ... ... Vol. XVII. [1902]

Progrès Médical, Le ... ... ... ... ... ... ... ... Tom. XV., XVI. 1902

Progressive Medicine ... ... ... ... ... ... ... ... ... Vol. IV. 1902

Publishers' Circular, The... ... ... ... (4) Vols. LXXVI., LXXVII. 1902

Revue d'Obstétricale et Gynécologie ... ... ... ... [Vol. XVIII.] 1902

Revue générale d'Ophtalmologie ... ... ... ... ... ... Tome XXI. 1902

Revue hebdomadaire de Laryngologie, d'Otologie et de Rhinologie. Tome XXII. 1902

St. Louis Medical and Surgical Journal, The Vols. LXXXII., LXXXIII. 1902

Sanitary Record, The ... ... ... ... ... ... ... ... ... Vol. XXIX. 1902

Sei-I-Kwai Medical Journal, The ... ... ... ... ... ... Vol. XXI. 1902

Therapeutic Gazette, The ... ... ... ... ... ... ... Vol. XXVI. 1902

Weiner klinische Wochenschrift ... ... ... ... ... ... ... ... ... 1902

### MEETINGS OF SOCIETIES.

### Bristol Medico-Chirurgical Society.

*March 11th, 1903.*

Mr. G. Munro Smith, President, in the Chair.

Dr. J. Odery Symes showed microscopical specimens of the blood obtained from a case of quartan malaria in Cyprus.

### Local Medical Notes.

---

**University College, Bristol.**—Messrs. C. W. W. James and C. A. Moore have obtained the M.R.C.S. and L.R.C.P. diplomas. Messrs. W. J. H. Pinninger and A. N. Thomas have passed the Intermediate M.B. Examination of the London University.

**Bristol Royal Infirmary.**—The following appointments have recently been made: W. McCowen, M.R.C.S., L.R.C.P., Junior House Surgeon; J. H. Hawes, M.B. Dunelm, Casualty Officer; N. Conolly, M.R.C.S., L.R.C.P., Resident Obstetric Officer.

**Bristol General Hospital.**—The following appointments have recently been made: Barclay J. Baron, M.D., Hon. Consulting Surgeon to the Throat and Nose Department; J. Lacy Firth, M.D., M.S., F.R.C.S., Surgeon to the Throat and Nose Department; Hedley Hill, M.D. (Brux.), M.R.C.S., L.R.C.P., Assistant Anæsthetist; C. Corfield, M.R.C.S., L.R.C.P., Assistant House Physician; J. W. Llewellyn, M.R.C.S., L.R.C.P., Assistant House Surgeon; H. Devine, M.R.C.S., L.R.C.P., Casualty House Surgeon.

**The Bristol Dispensary** —The report for 1902 shows that the work accomplished by the medical officers is very large. Since the Institution was founded the number of patients relieved has been 515,895, and the total number of midwifery cases has been 49,335. There are eight medical officers working in the different districts, and the number of patients attended during the year 1902 was 12,551. The Committee has to record the loss during the past year of two members who have taken much interest in the Dispensary for many years,—Alderman Bartlett and Mr. Thomas Davey, who succeeded Alderman Francis J. Fry in the offices of Treasurer and Chairman. Mr. Fenwick Richards has now been appointed to these offices.

**The Queen Victoria Jubilee Convalescent Home.**—The third annual report, for 1902, shows a large increase of the number of patients admitted—1,390, as against 1,017 in the year 1901, an increase of 37 per cent. The Home is therefore maintaining and increasing its usefulness, not only amongst the Infirmary and Hospital patients, who are admitted free, but the number of paying patients has increased from 87 to 155: these make a weekly contribution of ten shillings for their maintenance. The Governors have every reason to think that the management of the Home is what they wish it to be, and that the arrangements are much appreciated by the inmates.

